# *Trichoderma* cf. *asperellum* and plant-based titanium dioxide nanoparticles initiate morphological and biochemical modifications in *Hordeum vulgare* L. against *Bipolaris sorokiniana*

**DOI:** 10.1186/s12870-024-04785-3

**Published:** 2024-02-17

**Authors:** Rabab A. Metwally, Shereen A. Soliman, Hanan Abdalla, Reda E. Abdelhameed

**Affiliations:** Botany and Microbiology Department, Faculty of Science, Zagazig University, Zagazig, 44519 Egypt

**Keywords:** Antagonistic activity, Antioxidative enzymes, Barley, Biocontrol agent, Cell free filtrate, Green synthesis, Spot blotch disease, *Trichoderma* cf*. asperellum*

## Abstract

**Background:**

Spot blotch is a serious foliar disease of barley (*Hordeum vulgare* L.) plants caused by *Bipolaris sorokiniana,* which is a hemibiotrophic ascomycete that has a global impact on productivity. Some *Trichoderma* spp. is a promising candidate as a biocontrol agent as well as a plant growth stimulant. Also, the application of nanomaterials in agriculture limits the use of harmful agrochemicals and helps improve the yield of different crops. The current study was carried out to evaluate the effectiveness of *Trichoderma.* cf*. asperellum* and the biosynthesized titanium dioxide nanoparticles (TiO_2_ NPs) to manage the spot blotch disease of barley caused by *B. sorokiniana* and to assess the plant’s innate defense response.

**Results:**

*Aloe vera* L. aqueous leaf extract was used to biosynthesize TiO_2_ NPs by reducing TiCl_4_ salt into TiO_2_ NPs and the biosynthesized NPs were detected using SEM and TEM. It was confirmed that the NPs are anatase-crystalline phases and exist in sizes ranging from 10 to 25 nm. The *T.* cf. *asperellum* fungus was detected using morphological traits and rDNA ITS analysis. This fungus showed strong antagonistic activity against *B. sorokiniana* (57.07%). Additionally, *T.* cf. *asperellum* cultures that were 5 days old demonstrated the best antagonistic activity against the pathogen in cell-free culture filtrate. Also, *B. sorokiniana* was unable to grow on PDA supplemented with 25 and 50 mg/L of TiO_2_ NPs, and the diameter of the inhibitory zone increased with increasing TiO_2_ NPs concentration. In an in vivo assay, barley plants treated with *T.* cf. *asperellum* or TiO_2_ NPs were used to evaluate their biocontrol efficiency against *B. sorokiniana,* in which *T.* cf. *asperellum* and TiO_2_ NPs enhanced the growth of the plant without displaying disease symptoms. Furthermore, the physiological and biochemical parameters of barley plants treated with *T.* cf. *asperellum* or TiO_2_ NPs in response to *B. sorokiniana* treatment were quantitively estimated. Hence, *T.* cf. *asperellum* and TiO_2_ NPs improve the plant’s tolerance and reduce the growth inhibitory effect of *B. sorokiniana*.

**Conclusion:**

Subsequently, *T.* cf. *asperellum* and TiO_2_ NPs were able to protect barley plants against *B. sorokiniana* via enhancement of chlorophyll content, improvement of plant health, and induction of the barley innate defense system. The present work emphasizes the major contribution of *T.* cf*. asperellum* and the biosynthesized TiO_2_ NPs to the management of spot blotch disease in barley plants, and ultimately to the enhancement of barley plant quality and productivity.

## Key points

*• T.* cf. *asperellum* showed strong antagonistic activity against *B. sorokiniana invitro*.

*•* Plant- based synthesis of TiO_2_ NPs (10- 25 nm) using *Aloe vera* L. aqueous leaf extract.

*• B. sorokiniana* triggers morphological and biochemical changes in barley plants, causing spot blotch disease.

*• T.* cf. *asperellum* or green synthesized TiO_2_ NPs positively increased the host plant's tolerance against this disease by inducing of osmolytes and antioxidant defense-related enzyme production.

## Background

Barley (*Hordeum vulgare* L.) is the world's fourth most-produced cereal [1, 2], coming after wheat, rice, and corn. According to estimates by Triticase et al. [3], 21% of barley production was used in the malting and brewing industries, 70% went towards animal feed, and only roughly 6% was consumed by humans.

In agriculture, plant diseases contribute significantly to the depletion of natural resources and are thought to be a significant factor in global food production's yearly decline [4, 5]. In particular, soil-borne pathogens pose a serious threat, with fungi being the most active [6]. Due to changes in agricultural practices over the past few years, the proliferation of various phytopathogenic fungi, including *Fusarium* spp, *Rhizoctonia* spp, *Alternaria* spp, *Botrytis* spp, and *Helminthosporium* spp. has been damaging to crops with significant economic losses [7–12]. Barley is an excellent example of such crops that are exposed to various fungal pathogens, among which *Bipolaris sorokiniana* causes leaf blight (also known as spot blotch), black point, and other foliar and root diseases.

Spot blotch disease is one of the most serious diseases of barley and can reduce yield by more than 30% and have an impact on malting quality [13, 14]. It happens in warm, humid areas of the world. *B. sorokiniana* fungus may infect the plant's coleoptiles, crowns, culms, leaves, and roots [15]. Small brown patches that are first caused by spot blotches grow into dark brown blotches [16]. A zone of yellow leaf tissue with different widths that separates leaf spots may cause the production of shriveled seeds and reduce yield [14, 17]. Management of spot blotch disease has relied on the application of fungicides, cultural practices, disease-resistant cultivars, and biological control agents by beneficial microbes [18–20]. However, its management by beneficial microorganisms is a promising biocontrol strategy, as these beneficial microorganisms are essential for increasing nutrient availability, promoting plant development, combating soil-borne pathogens, and stimulating the plant's immune system [20–22].

Since they are considered symbiotic, opportunistic, and non-virulent, *Trichoderma* spp. have been used as biological control agents against plant pathogenic fungi instead of synthetic pesticides [23–25]. Their biological control strategies involve activating various processes, either indirectly through the competition for resources and space, the stimulation of plant growth and defense systems, or directly through mycoparasitism and antibiosis [26, 27]. Some *Trichoderma* spp. is associated with numerous plants through endophytic associations and can colonize the root surface [28]. Because of this symbiotic association, the plant is efficiently protected from pathogens [29, 30], where *Trichoderma* causes the expression of genes involved in plants' defensive mechanisms when it interacts with them [31, 32] and stimulates root development and plant growth [33–36]. For example, Morais et al. [27] investigated that *Trichoderma* spp. was used to assess the antagonistic activity against *Colletotrichum truncatum, Lasiodiplodia theobromae, Sclerotium delphinii,* and *Macrophomina phaseolina*. Also, *T. viride* and *T. harzianum* evidenced high efficiency against *A. alternata* and *Drechslera halodes* [37]*.* When employed to control *R. solani* and *F. oxysporum* f. sp. lycopersici in tomato plants, *T. atrobrunneum* and *T. simmonsii* significantly increased stem height and fresh weight in pathogen-treated tomato plants [38].

Another way to enhance the defense machinery of plants is through the use of modern technologies, i.e. nanotechnology, which can be extremely helpful in addressing this problem by restricting the use of damaging agrochemicals and assisting in boosting the yield of different crops [39, 40]. Nanoparticles (NPs) are recognized as a plant growth stimulant that modifies physiological, biochemical, and physicochemical pathways [41, 42]. Recently, titanium dioxide nanoparticles (TiO_2_ NPs), a type of metal oxide nanomaterial, have gained popularity as an environmentally friendly and clean photocatalyst due to their optical qualities, chemical stability, and non-toxicity [43–45]. These NPs have powerful oxidizing properties that produce free radicals like superoxide anion radicals, which inhibit the growth of microorganisms. As a result, they can be used in the agriculture industry to protect plants and inactivate various pathogenic infections [46].

By taking advantage of the reducing qualities of plant secondary metabolites, green synthesis of NPs can advantageously synthesize functional NPs [45, 47]. These benefits include producing biologically active nanomaterials, using inexpensive reactants, and having an environmentally favorable synthesis process [40]. TiO_2_ NPs made from plants have the potential to decrease the severity of diseases and stimulate plant growth by quickly absorbing on the surface of the plant and pathogens [48]. Currently, it is crucial to recognize the plant's defense mechanism against fungal pathogens. Few scientific papers have discussed the function of TiO_2_ NPs in reducing disease severity and their effects on plant biochemistry and productivity in response to fungal stress [49]. Hence, in this study, we attempted to go deeply into investigating the impact of *T.* cf*. asperellum* and green synthesized TiO_2_ NPs on the growth of barley plants and the severity of spot blotch disease caused by *B. sorokiniana*. As well, their effects on biochemical analysis and the defense-related enzymes in barley plants were also evaluated.

## Results

### Molecular identification of *Trichoderma sp.* and *Bipolaris sp.*

**Fig. 1:**
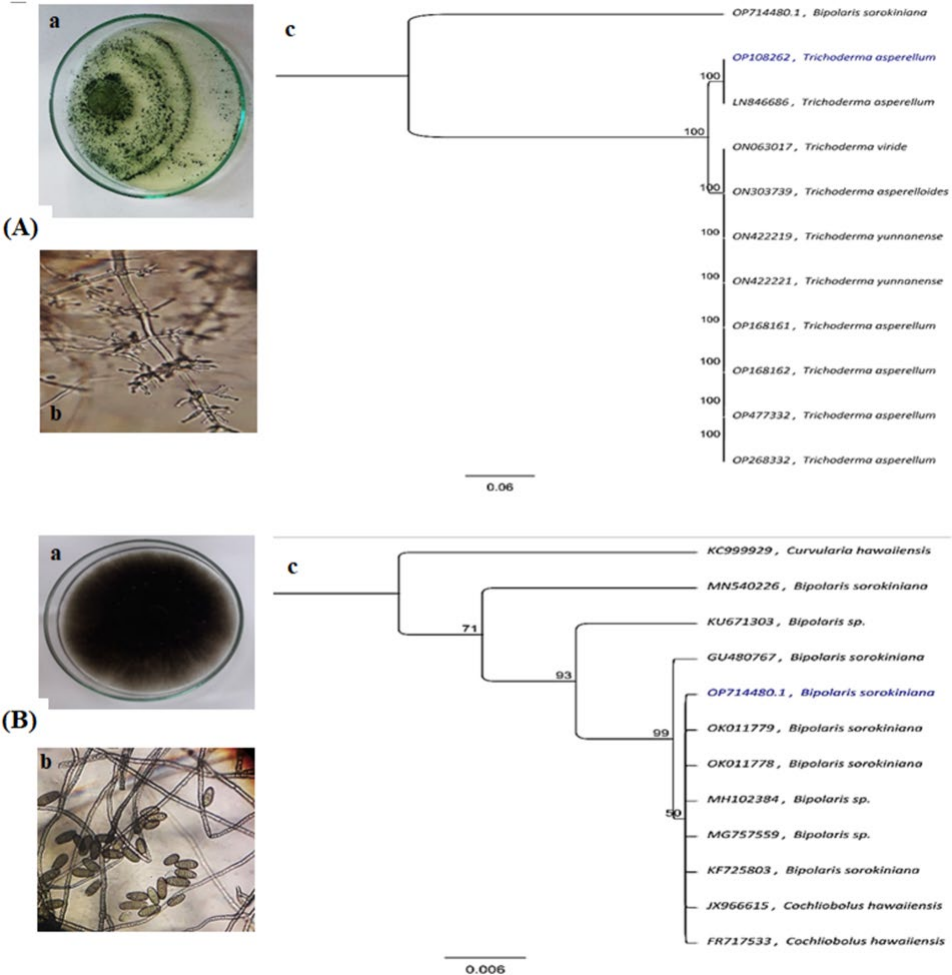
Morphological and molecular characterization of (**A**) *T.* cf. *asperellum* (biocontrol agent): (a) colony on PDA, (b) vegetative mycelium microstructure showing conidiophore and phialides, (c) phylogenetic tree (**B**) *B. sorokiniana* (pathogen): (a) colony on PDA, (b) vegetative mycelium microstructure showing hyphae and conidia, (c) phylogenetic tree

The morphological identification (Fig. 1) of *Trichoderma* sp. and *Bipolaris* sp. was verified through the use of ITS1/ITS4 primers in a molecular investigation of the ITS rDNA sequence (18S-28S rRNA), flanking ITS1 (5.8S rRNA), and ITS2 as *T.* cf. *asperellum* and *B. sorokiniana*. The retrieved ITS sequence was entered into NCBI GenBank as accession no.: OP108262 and OP714480 using the BLAST program, and in order to compare with other relevant strains, the sequence data was aligned. Phylogenetic analysis was performed using MEGA version 7 software, which stands for molecular evolutionary genetic analysis. Based on the ITS gene sequences, the phylogenetic tree displayed in Fig. 1c was constructed using the neighbor-joining method with 1000 bootstrap repetitions. Multiple sequence alignments were performed using the ClustalW programme in the MEGA7 software using the closest homologous sequences that were chosen. The maximum composite likelihood approach was used to compute the evolutionary distances.

### In vitro antifungal assay

**Fig. 2:**
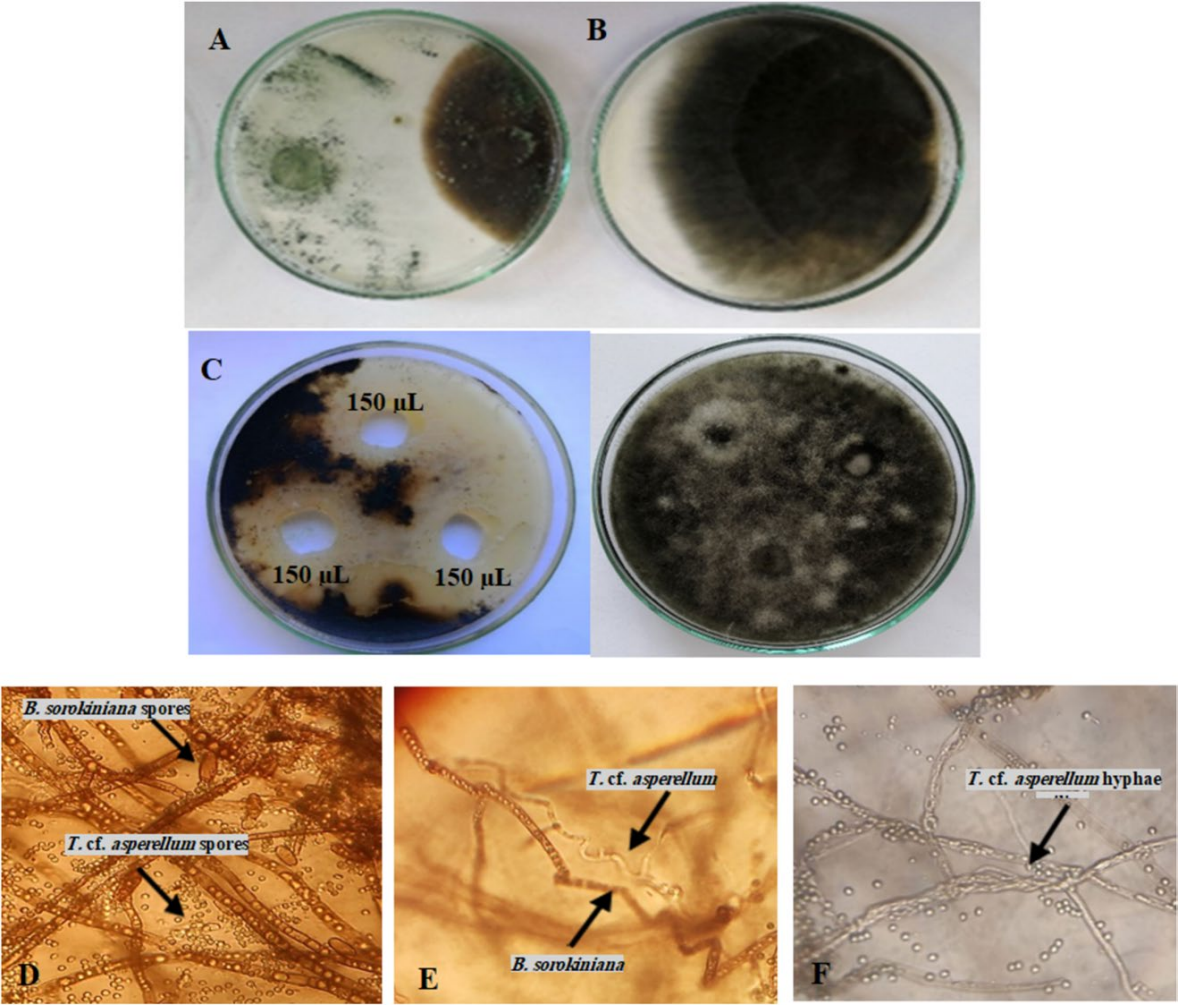
Dual culture for the antagonistic effect evaluation of *T.* cf. *asperellum* against *B. sorokiniana*. **A** *T.* cf. *asperellum* and *B. sorokiniana* interaction, (**B**) *B. sorokiniana* control, (**C**) In vitro control of *B. sorokiniana* using cell-free culture filtrate of (150 µL) by well diffusion method after 5 days of incubation. **D**-**F** Mycoparasitism of *T.* cf. *asperellum* against mycelium of *B. sorokiniana* (**D**) intensively vacuolated deformed mycelium of *B. sorokiniana*, (**E**) and (**F**) *T.cf. asperellum* and *B. sorokiniana* hyphal interaction

*T.* cf. *asperellum* was isolated from the rhizosphere of plants grown in the Egyptian soil and showed an inhibitory effect against *B. sorokiniana*. The dual culture test illustrated in Fig. 2A showed the inhibition zone between *B. sorokiniana* and *T.* cf*. asperellum*, where *T.* cf. *asperellum* exhibited strong antagonistic activity against *B. sorokiniana* (57.07%) that proved its potent activity as compared to *B. sorokiniana* (Fig. 2B). Also, Fig. 2 (C) showed the antagonistic effect of *T.* cf*. asperellum* cell-free culture filtrate against *B. sorokiniana*. Figure 2 (D-F) showed the mycoparasitism of *T asperellum* against mycelium of *B. sorokiniana,* intensively vacuolated deformed mycelium of *B. sorokiniana* and *T.* cf. *asperellum* and *B. sorokiniana* hyphal interaction.

### Characterization of TiO_2_ NPs' morphology and optics

**Fig. 3:**
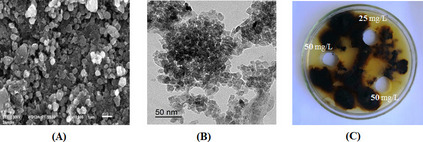
SEM (**A**) and TEM (**B**) images of green synthesized TiO_2_ NPs from *A. vera* leaf aqueous extract. **C** In vitro control of *B. sorokiniana* using the green synthesized TiO_2_ NPs (25 and 50 mg/L) from *A. vera* leaf aqueous extract by well diffusion method after 5 days of incubation

An early indicator of synthesis is the transformation of TiCl_4_ from a milky off-white to a reddish brown color after 4 h of stirring with *A. vera* aqueous extract. As shown in Fig. 3A and B, the SEM and TEM analysis of TiO_2_ NPs indicated that they are tetragonal in shape, with the majority of the nano-forms being found in the 10 to 25 nm size range.

### Antifungal activity of TiO_2_ NPs against *B. sorokiniana*

The well diffusion method was used to determine the effect of two different concentrations of green-synthesized TiO_2_ NPs against *B. sorokiniana*. The results are displayed in Fig. 3 (C). The results showed that on PDA agar plates treated with 25 and 50 mg/L of TiO_2_ NPs, *B. sorokiniana* was not able to grow and that the diameter of the inhibition zone increased with TiO_2_ NPs concentration. Based on these findings, a concentration of 50 mg/L showed a promising antifungal action against *B. sorokiniana*.

### Efficacy of *T. cf. asperellum* on growth attributes of barley against *B. sorokiniana* in response to TiO_2_ NPs

**Fig. 4:**
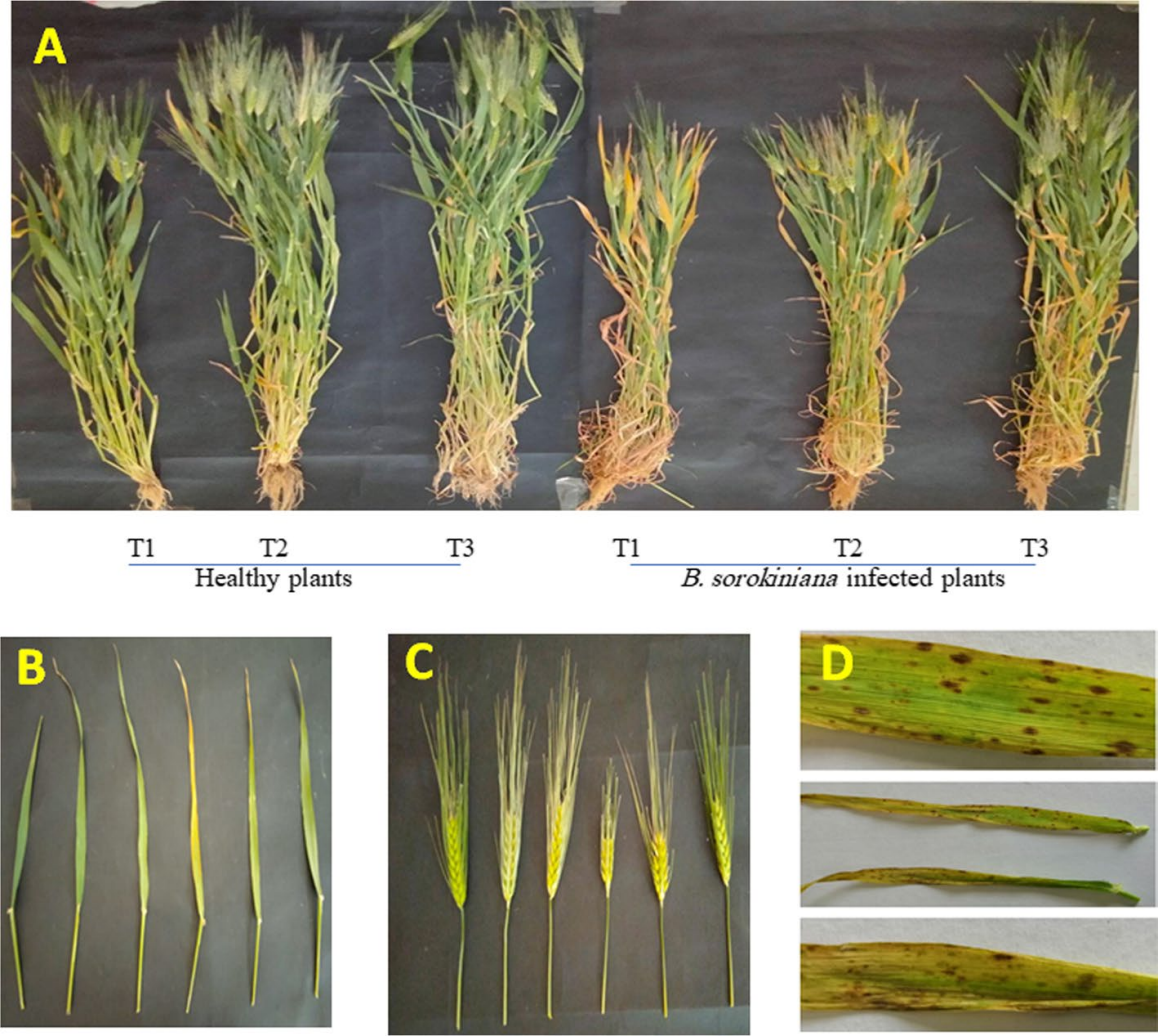
In vivo effect of *T.* cf. *asperellum* and TiO_2_ NPs against *B. sorokiniana* infection on barley plants (**A**), barley leaves (**B**), barley spikes (**C**) and (**D**) leaf spot blotch on barley leaves after 4 weeks from *B. sorokiniana* infection. T1, T2 and T3 refers to control, TiO_2_ NPs and *T. cf. asperellum* treated plants respectively

Infected barley plants with *B. sorokiniana* showed a decline in their morphological attributes (Fig. 4A-C). Figure 4(D) showed typical spot blotch symptoms on barley plant leaves challenged with *B. sorokiniana*. The barley height, shoot and root fresh weight (fwt), and dry weight (dwt) (Table 1) were significantly reduced compared to the healthy control. However, *T.* cf. *asperellum* significantly increased the barley growth parameters compared to the *B. sorokiniana*- infected plants and also showed a reduction in disease symptoms caused by *B. sorokiniana*. *T.* cf. *asperellum* increased barley height, shoot, and root fwt by 33.3, 20.7, and 34%, respectively. Generally, barley plants diseased with *B. sorokiniana* exhibited a significant decrease in spike fwt and dwt (Fig. 4C and Table 1) reaching 62.9 and 57.9% respectively, compared to control plants. Moreover, the data in Table 1 also revealed that the biosynthesized TiO_2_ NPs had a positive impact on the growth parameters of barley and improved both fwt and dwt. The percentage enhancement due to TiO_2_ NPs application of shoot fwt and dwt of *B. sorokiniana*- infected plants was 30 and 31.4 as compared to *B. sorokiniana*-infected ones.

**Table 1:** Effect of *T.* cf. *asperellum* and TiO_2_ NPs on the plant growth of barley infected with *B. sorokiniana* pathogen, and uninfected

Parameters	Shoot height (cm)	Shoot fwt (g)	Root fwt (g)	Shoot dwt (g)	Root dwt (g)	Spike fwt (g)	Spike dwt (g)
Treatments							
Control	30 ± 0.794b	13.97 ± 0.369b	1.130 ± 0.0299c	4.030 ± 0.106b	0.447 ± 0.012c	3.7 ± 0.098c	1.38 ± 0.036b
*B. sorokiniana*	24 ± 0.635d	9.10 ± 0.241d	0.607 ± 0.016d	2.987 ± 0.079c	0.220 ± 0.0058e	1.37 ± 0.036f	0.580 ± 0.015d
TiO_2_ NPs	38 ± 1.01a	15.90 ± 0.421a	1.407 ± 0.037b	4.729 ± 0.125a	0.662 ± 0.017b	4.1 ± 0.108b	1.428 ± 0.038ab
TiO_2_ NPs + *B. sorokiniana*	27 ± 0.714c	11.83 ± 0.313c	1.060 ± 0.028c	3.927 ± 0.104b	0.376 ± 0.0099d	2.24 ± 0.059e	0.962 ± 0.025c
*T.* cf*. asperellum*	40 ± 1.06a	16.87 ± 0.446a	1.517 ± 0.041a	4.967 ± 0.131a	0.753 ± 0.019a	4.5 ± 0.119a	1.498 ± 0.039a
*T.* cf. *asperellum* + *B. sorokiniana*	28 ± 0.741bc	13.04 ± 0.345b	1.133 ± 0.029c	4.177 ± 0.111b	0.481 ± 0.0127c	2.89 ± 0.076d	1.051 ± 0.028c

Values are the mean ± standard error of the mean (*n* = 5). Data analysis was done by using Duncan’s multiple range test at *p* ≤ 0.05. The same letters within a column are not significantly different

### Evaluation of barley physio-biochemical characteristics in response to *T. cf. asperellum* and TiO_2_ NPs application to *B. sorokiniana* stress

Barley leaves were tested for the presence of photosynthetic pigments (Chl a, Chl b, and total Chl), which revealed a severe deficiency in their contents of 26.7, 19.58, and 24.67%, respectively, in *B. sorokiniana* pathogen-challenged plants compared to controls. Interestingly, treating diseased or healthy plants with *T.* cf. *asperellum* or TiO_2_ NPs led to a noticeable improvement in Chl pigments. When compared to healthy plants, *T.* cf*. asperellum* had the highest levels of Chl a, Chl b, and carotenoids (13.9, 8.56, and 42.7%, respectively) in its photosynthetic pigment content (Table 2). Also, barley plants treated with TiO_2_ NPs also displayed improved levels of Chl a, Chl b, and total Chl. In plants under stress, the total Chl content increased in those exposed to TiO_2_ NPs from 1.523 to 2.082 mg/g leaf fwt.

**Table 2:** Influence of *T.* cf. *asperellum* and TiO_2_ NPs on photosynthetic pigments (mg g^−1^ leaf fwt) of barley plants infected with *B. sorokiniana* pathogen, and uninfected

Parameters Treatments	**Chl a**	**Chl b**	**Total chlorophylls**	**Carotenoids**	**Total pigments**	**Chl a/Chl b**
Control	1.450 ± 0.038b	0.572 ± 0.015b	2.022 ± 0.053b	0.737 ± 0.019c	2.759 ± 0.073c	2.534 ± 0.067a
*B. sorokiniana*	1.063 ± 0.028d	0.460 ± 0.012c	1.523 ± 0.04d	0.716 ± 0.019c	2.240 ± 0.059d	2.312 ± 0.061b
TiO_2_ NPs	1.501 ± 0.039b	0.581 ± 0.0153ab	2.082 ± 0.055b	0.905 ± 0.024b	2.987 ± 0.079b	2.583 ± 0.068a
TiO_2_ NPs + *B. sorokiniana*	1.137 ± 0.031cd	0.441 ± 0.011c	1.578 ± 0.042cd	0.702 ± 0.018c	2.280 ± 0.06d	2.578 ± 0.068a
*T.* cf.* asperellum*	1.652 ± 0.044a	0.621 ± 0.0164a	2.273 ± 0.06a	1.052 ± 0.027a	3.325 ± 0.087a	2.660 ± 0.07a
*T.* cf*. asperellum* + *B. sorokiniana*	1.217 ± 0.032c	0.485 ± 0.013c	1.702 ± 0.045c	0.739 ± 0.0195c	2.441 ± 0.064d	2.507 ± 0.066ab

Values are the mean ± standard error of the mean (*n* = 5). Data analysis was done by using Duncan’s multiple range test at *p* ≤ 0.05. The same letters within a column are not significantly different

**Fig. 5:**
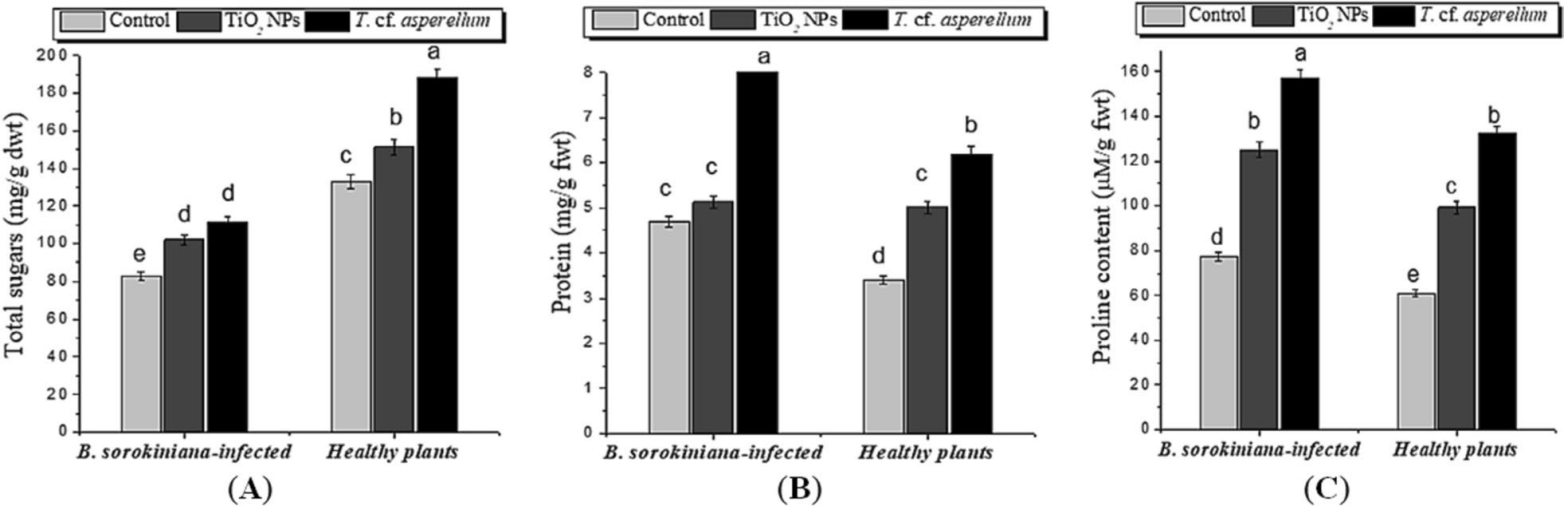
Influence of *T.* cf. *asperellum* and TiO_2_ NPs on osmolytes: (**A**) total sugars, (**B**) protein and (**C**) proline of barley plants infected with *B. sorokiniana* pathogen, and uninfected. Data analysis was done by using Duncan’s multiple range test at *p* ≤ 0.05. The same letters are not significantly different and error bars reflect the standard error of the mean

In terms of total sugar contents (Fig. 5A), barley plants infected with *B. sorokiniana* showed a marked reduction in their amount (83.133 ± 2.199 mg/g dwt) as compared to uninfected plants (132.86 ± 3.52 mg/g dwt). Spraying *T.* cf. *asperellum* or TiO_2_ NPs had a notable and beneficial impact on total sugars in both healthy and *B. sorokiniana*-stressed plants (Fig. 5A, Table 3). Moreover, the data in Table 4 revealed a positive Pearson’s correlation between total sugars and pigment fractions (Chl a ‘0.974’, Chl b ‘0.926’, and total pigments ‘0.983’). The results in Fig. 5B and C also revealed a rise in the total protein and proline content in *B. sorokiniana***-**infected plants compared to the healthy plants (negative control). However, barley plants treated with *T.* cf. *asperellum* and infected with *B. sorokiniana* showed further proliferation in their contents (6.194 ± 0.164 mg/g fwt and 132.09 ± 3.49 µmol/g fwt) as compared with the infected ones (4.854 ± 0.128 mg/g fwt and 77.28 ± 2.04 µmol/g fwt), and the same trend was reported for TiO_2_ NPs.

**Table 3:** Analysis of variance (Two-way ANOVA) of the effect of *T.* cf*. asperellum*, TiO_2_ NPs, *B. sorokiniana* pathogen and their interactions on some morpho-biochemical parameters of barley plants

	***T.* cf.* asperellum***	**TiO**_**2**_** NPs**	***B. sorokiniana***	***T.* cf.* asperellum*** ** + ** ***B. sorokiniana***	***B. sorokiniana* + ** **TiO**_**2**_** NPs**
Shoot height	*	*	*	*	*
Root fwt	*	*	*	*	ns
Shoot fwt	*	*	*	*	*
Chl a	*	*	*	*	*
Chl b	*	*	*	*	*
H_2_O_2_	*	*	*	*	*
MDA	*	*	*	*	*
Protein	*	*	ns	*	*
Total sugars	*	*	*	*	*
Proline	*	*	*	*	*
CAT	*	*	*	*	*
POX	*	ns	*	*	*
PAL	*	*	*	*	*

*fwt* fresh weight, *MDA* malondialdehyde

^*^Significant at the* p* < 0.05; *ns* non-significant

**Table 4:** Pearson’s correlation matrix between growth parameters of barley plants (shoot height, shoot fwt and root fwt)), photosynthetic pigments (Chl a, Chl b and total pigments) and other biochemical parameters (total sugars, MDA and H_2_O_2_). Each square indicates the Pearson’s correlation coefficient of a pair of parameters

Measured parameters	Shoot height	Shoot fwt	Root fwt	Chl a	Chl b	Total pigments	Total sugars	MDA	H_2_O_2_
Shoot height	1.000								
Shoot fwt	0.951**	1.000							
Root fwt	0.921**	0.981**	1.000						
Chl a	0.939**	0.952**	0.882**	1.000					
Chl b	0.896**	0.889**	0.786*	0.981**	1.000				
Total pigments	0.964**	0.935**	0.867**	0.987**	0.972**	1.000			
Total sugars	0.964**	0.956**	0.917**	0.974**	0.926**	0.983**	1.000		
MDA	-0.844**	-0.940**	-0.939**	-0.864**	-0.782*	-0.862**	-0.888**	1.000	
H_2_O_2_	-0.887**	-0.900**	-0.898**	-0.868**	-0.783*	-0.825**	-0.930**	0.949**	1.000

*fwt* fresh weight, *MDA* malondialdehyde

^*^Correlation was significant at the* p* < 0.05

^**^Correlation was significant at the *p* < 0.01

### Reduction of oxidative burst and lipid peroxidation

**Fig. 6:**
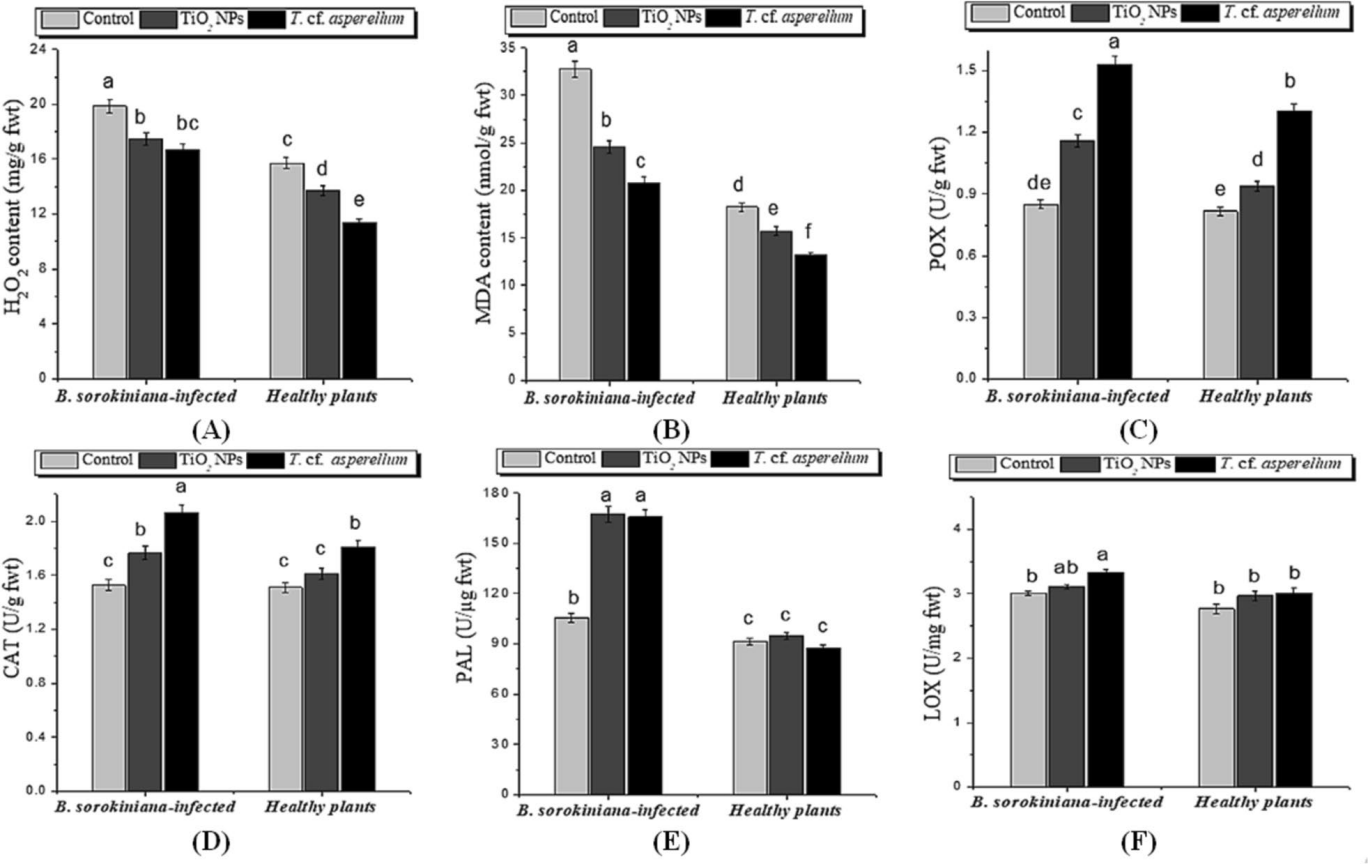
Effect *of T.* cf. *asperellum* and TiO_2_ NPs on stress markers: (**A**) H_2_O_2_, (**B**) Malondialdehyde (MDA), (**C**) POX, (**D**) CAT, (**E**) PAL and (**F**) LOX of barley infected with test *B. sorokiniana* pathogen, and uninfected. Data analysis was done by using Duncan’s multiple range test at *p* ≤ 0.05. The same letters are not significantly different and error bars reflect the standard error of the mean

The findings presented in Fig. 6 (A and B) demonstrated that, in comparison to healthy plants, the *B. sorokiniana* infection of barley plants resulted in a notable and elevated accumulation of H_2_O_2_ and MDA content (26.33 and 79.56%, respectively). It is important to note that *T.* cf*. asperellum* caused the least reduction in lipid peroxidation and H_2_O_2_ concentration*.* The application of *T.* cf. *asperellum* or TiO_2_ NPs retarded the values of H_2_O_2_ (27.8 and 12.5%) and lipid peroxidation (27.9 and 13.78%) as compared to healthy plants (Fig. 6). Moreover, the elevated concentration of H_2_O_2_ in the cellular system positively and significantly correlates (Table 4, *r* = 0.949**) with the oxidative changes affecting MDA content.

### Enhancing antioxidant and defense related enzymes activity

POX, CAT, and PAL activities increased in barley plant leaves challenged with *B. sorokiniana* (42, 17, and 73%) as compared to untreated negative control plants. Remarkably, based on ANOVA results, barley plants sprayed with *T.* cf. *asperellum* or TiO_2_ NPs showed a significant increase in their activities over pathogen-challenged plants (Fig. 6C-E and Table 3). Furthermore, an increase in LOX activity (Fig. 6F) was observed in barley plants infected with *B. sorokiniana*. Furthermore, under pathogen-challenged conditions, treatment with *T.* cf. *asperellum* or TiO_2_ NPs slightly and non-significantly increased LOX levels in plant leaves when compared to *B. sorokiniana*-treated plants and untreated control plants.

## Discussion

The hemibiotrophic ascomycete *B. sorokiniana* poses a major danger to the production of barley and other cereal crops. Numerous chemical fungicides have been applied, but since the disease is so sophisticated, the pathogens have evolved to become resistant to these chemicals [50]. Therefore, biological management may be a good choice for high quality and productivity in crop production and sustainable agriculture. Recently, biocontrol agents that employ bacteria and fungi have drawn a lot of interest as a secure and effective disease prevention strategy. To do this, scientists have looked into a variety of microorganisms, including fungi and bacteria like *Chaetomium globosum*, *T. reesei, T. hamatum, T. harzianum* and *Bacillus subtilis *TE3, to manage the spot blotch pathogen [51–53].

It is obvious that *T.* cf*. asperellum* showed an inhibitory effect against *B. sorokiniana*. Our results of the antifungal activity of *T.* cf. *asperellum* against *B. sorokiniana* agree with Yassin et al. [37], Matroudi et al. [54] and Mukherjee et al. [55] who studied the efficacy of *T.* cf. *asperellum* against a wide range of soil-borne fungi due to the production of antifungal metabolites. Druzhinina et al. [26] and Morais et al. [27] confirmed that the mycoparasitism and antibiosis attributes of some *Trichoderma* spp. in addition to competition for resources and space, are used by humans for biological control activities. Furthermore, *T.* cf. *asperellum* induces morphological modifications that allow it to penetrate the host and hold high quantities of osmotic solutes such as glycerol [55, 56]. *Trichoderma* attaches to the pathogen through cell-wall carbohydrates that bind to the lectins on the pathogen [57]. The creation of cell wall-degrading enzymes and peptaibols [58] is the next stage, which makes it easier for *Trichoderma* hypha to enter the parasitized fungus' lumen and for the cell-wall content to be assimilated.

The one-step reaction, environmentally friendly reactants, and cost-effectiveness of the green NPs synthesis make it superior to the chemical and physical approaches. As a result, biocompatible active NPs with several biological and medicinal uses are created [40]. Plant secondary metabolites are important for reducing and stabilizing bulk materials in redox processes during the creation of NPs [59]. *A. vera* leaf aqueous extract was qualitatively assessed, as earlier mentioned in our previous study [60] and the results showed the presence of some phytochemicals such as phenolics, flavonoids, alkaloids, and tannins. In this study, TiO_2_ NPs were prepared by green synthesis at pH 9 using phytochemicals in *A. vera* extract to reduce the TiCl_4_ salt into TiO_2_ NPs. An initial sign of synthesis is the change of the milky off-white color of TiCl_4_ to a pinkish brown color after 4 h of stirring with *A. vera* aqueous extract. Visual observation of this color change was in line with results presented in Satti et al. [49]. As previously described in our study, according to XRD results, the synthesized TiO_2_ NPs were pure and formed of only the anatase crystalline phase [60]. Burda et al.'s study [61] confirmed that the two factors affecting TiO_2_ NPs' physicochemical properties and consequently their antibacterial properties are their shape and crystal structure. More specifically, anatase has the highest levels of photocatalytic and antibacterial activity. The anatase structure is capable of producing OH˙ radicals during a photocatalytic event, which can destroy the pathogen membranes. Using a leaf extract from *Trigonella foenum-graecum*, Gomathipriya and Subhapriya [62] biosynthesized TiO_2_ NPs, producing spherical NPs with diameters ranging from 20 to 90 nm. In this study, we evaluated the potential of synthetic TiO_2_ NPs as fungal pathogen biocontrol agents.

The use of biosynthesized metal NPs to fight fungi that cause plant diseases has increased recently [63–66]. The results showed that *B. sorokiniana* was unable to grow on PDA agar plates that had been supplemented with 25 and 50 mg/L of TiO_2_ NPs; in addition, the diameter of the inhibitory zone increased with increasing TiO_2_ NPs concentration. Our findings concur with those of Boxi et al. [67] and Irshad et al. [68] that hazardous plant pathogens such as *F. solani, Venturia inaequalis*, and *Ustilago tritici* were resistant to the strongest antifungal effects of TiO_2_ NPs. TiO_2_ NPs can induce cell damage in *Pichia pastoris* by impairing the ROS-associated scavenging system, which results in the accumulation of ROS [69]. Additionally, these NPs have the ability to penetrate cells and destroy the fungal cell wall. However, their entry into the cell, diffusion, and endocytosis might cause the creation of ROS, which can impair the performance of numerous intracellular organs [64, 70, 71]. By causing oxidative stress, ROS appears to play a significant role in the antifungal response. Additionally, they can destroy all cellular macromolecules, such as DNA and proteins [63]. NPs can also cause fungal death by disrupting cellular enzymes and interfering with the electron transfer chain [66].

In a field experiment, the antifungal properties of *T.* cf*. asperellum* and TiO_2_ NPs against the biotic stress of the spot blotch disease were investigated. The morphological traits of *B. sorokiniana*-infected barley plants decreased. However, adding biosynthesized TiO_2_ NPs or *T.* cf. *asperellum,* a fungus that promotes plant growth, greatly improved the growth parameters for barley plants. Our results are in agreement with Khalil et al. [72] results that the foliar application of *T. viride* and *C. globosum* either alone or in combination increased tomato fwt and dwt of shoots and roots as compared to control. Also, Abdelhameed and Metwally [25] reported that *T. viride* enhanced onion plant growth under normal conditions. *T.* cf. *asperellum* colonization of cucumber roots has been demonstrated to improve the availability of P and Fe to plants, resulting in appreciable increases in dwt, shoot length, and leaf area [73]. Additionally, auxins made by *Trichoderma* spp. can promote plant growth and root formation [74].

Under *B. sorokiniana* stress, Satti et al. [49] demonstrated that the biosynthesized TiO_2_ NPs improved the agro-morphological traits (fwt and dwt) and yield parameters of wheat plants. Additionally, it was noted that applying TiO_2_ NPs improved *Zea mays* growth characteristics [75]. Mishra et al. [76] state that TiO_2_ NPs may regulate the activity of N metabolism-related enzymes, which facilitates plant uptake of more nutrients as these NPs convert N_2_ into organic nitrogen in the form of proteins and chlorophyll pigments [77], which in turn raises plant biomass and dwt. A study by Jaberzadeh et al. [78] stated that the foliar application of TiO_2_ NPs to wheat plants shows a rise in starch and gluten contents, which in turn boosts yield due to increasing *rubisco* activity. Also, Rizwan et al. [79] reported an increase in soybean yield after treating with TiO_2_ NPs because of the increased absorption of water by the plants.

The physio-biochemical properties of the barley plants were examined in order to investigate the antifungal effects of *T.* cf. *asperellum* and the biosynthesized TiO_2_ NPs against spot blotch disease in barley plants. Photosynthesis plays an essential role in plant productivity and takes place in green leaves [80]. A severe deficiency in the photosynthetic pigment contents in *B. sorokiniana* pathogen-challenged plants was detected. This might be due to the plant's failure to capture sunlight and the breakdown of Chl pigments and thus photosynthesis will be diminished [81, 82]. It is interesting that a noticeable improvement in Chl pigments appeared with *T.* cf. *asperellum* or TiO_2_ NPs applications. Our results are consistent with Khalil et al. [72] whose work showed that *T. viride* and *C. globosum* foliar application, either alone or in combination, exhibited a considerable increase in Chl contents but a decrease in H_2_O_2_ and MDA. The findings of our study also support those of Aldinary et al. [83], who found that the use of fungal endophytes increases the efficiency of photosynthesis due to numerous changes in the chloroplasts and contents of carotene and Chl, as *Trichoderma* increases gene expression regulating Chl biosynthesis, light-harvesting complex proteins, or Calvin cycle components. Similarly, Satti et al. [49] found that the spot blotch in wheat plants caused by *B. sorokiniana* resulted in a significant decline in all pigment fractions. Nevertheless, with the application of 40 mg/L TiO_2_ NPs, an improvement in these pigment fractions appeared. Morteza et al. [75] and Khodakovskaya and Lahiani [84] showed a significant increase in all pigment fractions with TiO_2_ NPs application, where TiO_2_ NPs increase plant growth and photosynthesis rate by producing more carbohydrates. Additionally, Rodrìguez-González et al. [85] noted that the photocatalytic capability of TiO_2_ NPs, which degrade many pesticide types, might be important for protecting plants from diseases since they do not form poisonous or toxic chemicals, leading to a high pathogen disinfection capacity [86]. We hypothesized that the increase in Chl content would have a significant effect on the rate of photosynthesis, enhance the productivity of carbohydrates, and ultimately result in an increase in fwts and dwts.

In order to overcome the negative effects of spot blotch disease caused by *B. sorokiniana* on morphological and Chl content, barley plants activate dual defense, which is characterized by the enhanced accumulation of different osmolytes (sugars, proteins, and proline) and increased antioxidant and defense-related enzymes (POX, CAT, PAL, and LOX). Sugars participate in physiological processes related to plant growth and development and are also involved in the response to a number of stresses, acting as nutrient and metabolite signalling molecules [87, 88]. A sharp decrease in their amount was recorded in barley plants infected with *B. sorokiniana* compared to uninfected, and with the applications of *T.* cf. *asperellum* or TiO_2_ NPs a significant and positive effect on total sugars was detected. These results agree with those of Khodakovskaya and Lahiani [84], in which TiO_2_ NPs produced more carbohydrates, thus promoting growth and photosynthesis rates in plants. Sugars act as osmotic agents, helping maintain plasma membrane integrity [87]. In addition, a study by Abdelhameed and Metwally [25] supported the idea that *T. viride* contributes to the rise in sugar levels in onion plants. This might be explained by the enhancing effect of *T.* cf. *asperellum* and TiO_2_ NPs in raising Chl concentration (seen above), which had a favorable influence on photosynthetic rate and led to an increase in the production of soluble sugars, boosting fwt and dwt. Furthermore, Ze et al. [89] suggested that TiO_2_ NPs may enhance light harvesting complex II gene expression in chloroplasts, which is consistent with our finding that TiO_2_ NP supplementation results in a rise in the concentration of soluble sugars.

Concerning the results of the total protein and proline content, there was an increase in their contents in *B. sorokiniana***-**infected plants compared to the healthy plants. However, barley plants treated with *T.* cf. *asperellum* or TiO_2_ NPs and infected with *B. sorokiniana* showed further proliferation in their contents. Our findings are consistent with those of Khodakovskaya and Lahiani [84], who found significant differences in the amounts of amide and carbohydrates in cucumber plants treated with TiO_2_ NPs, suggesting that TiO_2_ NPs can affect cucumber at the macromolecular level. Also, TiO_2_ NPs transform N_2_ into organic nitrogen in the form of proteins, which eventually results in an increase in protein content. Moreover, *T. viride* caused a substantial increase in protein content in onion plants [35]. Similar to this, it has been noted that proline content increased in *A. solani-*infected eggplant and tomato [12, 90]. Our findings are consistent with Satti et al.'s [49] observation that spot blotch stress led to significant increases in proline concentration in wheat plants. Furthermore, it was discovered that applying TiO_2_ NPs increased the proline content of the infected wheat plants. In the same respect, TiO_2_ NPs were proven to increase proline content in broad bean plants under both normal and abiotic stress conditions by Abdel Latef et al. [91]. In addition to maintaining cell turgor or osmotic balance, accumulating proline also stabilizes membranes to avoid electrolyte leakage and scavenges ROS to prevent protracted oxidative bursts in plants [92–94]. Lipids are crucial for preserving the structural integrity of cells. MDA is thought to be the most thiobarbituric acid-reacting molecule that demonstrates the extent of oxidative stress as a result of lipid peroxidation [93, 95, 96]. Infection of barley plants with *B. sorokiniana* induced a significant and high accumulation of H_2_O_2_ and MDA content. It is worth mentioning that the application of TiO_2_ NPs or *T.* cf. *asperellum* reduced the amount of H_2_O_2_ and lipid peroxidation. Similar to this, Khalil et al. [72] reported that the application of *T. viride* controlled the production of H_2_O_2_ and lipid peroxidation, maintaining cell homeostasis in tomato plants that were not infected by the pathogen. In accordance with these results, Abdelrhim et al. [97] also showed an augmentation of H_2_O_2_ and lipid peroxidation in wheat plants challenged with *R. solani* and the role of SiO_2_ NPs in reducing their contents and inducing pathogen disease resistance. As well, Abdalla et al. [60] showed a great reduction in the amounts of H_2_O_2_ and lipid peroxidation in soybean plants treated with TiO_2_ NPs. Moreover, the elevated concentration of H_2_O_2_ in the cellular system positively correlates with the oxidative changes affecting MDA content, as it is formed by the reaction of ROS (H_2_O_2_ or/and O^−2^) with lipid molecules [98]. Soliman et al. [99] have reported that infection with *A. alternata* increased the amount of lipid peroxidation in the pathogen-inoculated pepper leaf samples.

Antioxidant enzymes have a crucial function in scavenging ROS and preventing the oxidative stress that leads to harmful effects on many sensitive molecules [100–103]. In general, POX, CAT, and PAL activities increased in barley plant leaves infected with *B. sorokiniana* and there was a further increase in their activities with *T.* cf. *asperellum* or TiO_2_ NPs application. It is known that *B. sorokiniana* stimulates cereals to activate a variety of secondary metabolic pathways [104]. Bagy et al. [105] reported that POX, which catalyzes H_2_O_2_ breakdown, is also implicated in lignification and suberization processes, which reduce pathogenesis and aid in infection prevention. Also, Kaur et al. [14] and Singla et al. [106] stated that PAL, the primary enzyme that connects primary to secondary metabolism, has been linked to the activation of responses against pathogenic fungi in barley. It is also involved in the synthesis of plant secondary antimicrobial substances that are essential for plant disease resistance and plays an essential role in the biosynthesis of lignin precursors [107]. Furthermore, Khalil et al. [72] revealed a substantial enhancement of CAT enzyme activity with *T. viride* and *C. globosum* applications. According to Naz et al. [108], a botanical chemical formulation caused cereals to become resistant to the fungus *B. sorokiniana* by activating POX and PAL enzymes. Similarly, wheat developed stress tolerance against this fungus by upregulating defense-related enzymes, including CAT, ascorbate peroxidase, PAL, and POX [52]. Moreover, Abdelrhim et al. [97] showed that the activity of PAL was intensified as SiO_2_ NPs were applied in wheat plants against *R. solani*. Kaur et al. [107], Ferrer et al. [109], Król et al. [110] and Abdelhameed et al. [111] reported that PAL and POX enzymes might serve as markers of induced resistance to fungal diseases. Related study by Metwally and Abdelhamed [112] revealed that NPs could likely boost POX and CAT activities, which directly contribute to overcoming various stresses.

Also, an increase in LOX activity was observed in barley plants infected with *B. sorokiniana*. Furthermore, treatment with *T.* cf. *asperellum* or TiO_2_ NPs increased LOX levels non-significantly. In the same trend, Ohta et al. [113] found an increase in LOX activity in rice leaves after infection with rice blast fungus, which is correlated with plant resistance against pathogens. As well, Nandini et al. [114] showed an increase in LOX and POX activities in pearl millet plants infected with downy mildew disease using crude proteins extracted from six different *Trichoderma* spp. This was attributed to the function of LOX in the establishment of the hypersensitive response, a form of programmed cell death that serves as an active defense mechanism [115, 116]. A necrotic lesion develops as a result of the rapid death of plant cells in the area surrounding the infection site, which stops the pathogen from spreading and causes more damage to the surviving plant organ.

## Methods

### In vitro experiments

#### Isolation of fungal pathogen (*Bipolaris sp.*)

The pathogenic fungus from diseased leaves of wheat and barley plants grown in the Egyptian soil of Minia Al-Qamh, El-Sharkia Governorate (30°31′25.4"N 31°21′13.1"E), showing typical spot blotch disease symptoms, was collected in paper bags. Isolation and purification of fungal pathogens were done according to Kumar et al. [117]. The infected wheat and barley plants' leaves were chopped into small pieces and surface sterilized in a 5% sodium hypochloride solution for 5 min, then rinsed in sterile distilled water. After drying in a sterile filter, the plant tissues were incubated for 6 days at 27°C in a potato dextrose agar (PDA) medium. To obtain pure cultures of the pathogen, colonies of the fungus that appeared were transferred to fresh PDA plates.

#### Isolation of biocontrol agent (*Trichoderma sp.*)

In the Egyptian soil of Minia Al-Qamh, El-Sharkia Governorate (30°31′25.4"N 31°21′13.1"E), *Trichoderma* sp. was isolated from barley plant rhizospheres using a serial dilution plate method. One mL of the suspension from each dilution was added aseptically into sterile Petri dishes filled with Rose Bengal medium. Three days later, *Trichoderma* colonies were selected and grown on PDA media. Pure isolates were made by inoculating the fungal colonies and letting them grow for seven days at 28°C. *Trichoderma* sp. stock culture was kept viable on PDA slants.

### Identification of fungal inoculants

#### Morphological identification of isolated fungi

By contrasting macroscopic characteristics on agar plates with those of microscopic aspects like hyphal branching pattern and conidial shape, the morphological characteristics of hyphae of *Trichoderma* sp. and *Bipolaris* sp*.*, were confirmed [118, 119] respectively. Figure 1 illustrates the morphological growth of both fungal inoculants of *Trichoderma* sp. (Fig. 1A [a and b]) and *Bipolaris* sp. (Fig. 1B [a and b]) on PDA plates.

#### Molecular identification of isolated fungi

The pure isolates of *Trichoderma* sp. and *Bipolaris* sp. were sub-cultured on a PDA medium and grown for 5 days at 27°C, and their genomic DNA was isolated using the CTAB technique [120]. The cell walls of fungal mycelia were broken down in the presence of liquid N_2_ by grinding. After that, the CTAB extraction buffer was added, and the mixture was incubated at 65°C before being purified using phenol, chloroform, and isoamyl alcohol (25:24:1). Cold isopropanol was used to precipitate the genomic DNA, which was then washed twice with cold 70% ethyl alcohol. Finally, the DNA was dissolved in 50 μL of sterilized distilled water.

#### PCR Amplification and phylogenetic analysis

The ITS1 and ITS2 as well as the inverted 5.8S coding rDNA were amplified using the ITS1 and ITS4 primers. In a total volume of 50 µL, each PCR reaction mixture comprised 5–10 ng of genomic DNA, 1 µM of each ITS1/ITS4 primer, 5 µL of a 10X reaction buffer (50 mM KCl, 50 mM Tris–HCl; pH 8.3, 0.1 mg/mL bovine serum albumin (BSA), 3 mM MgCl_2_, 200 µM each of dNTP, and 2.5 U of Taq DNA polymerase (Promega, Mannheim, Germany). The PCR technique includes 35 cycles of denaturation at 95°C for 30 s, annealing at 56°C for 30 s, and elongation at 72°C for 1 min. Before DNA sequencing, the PCR amplicon was resolved using an 8% agarose gel and purified using a specific PCR purification kit (Accu Prep® PCR DNA Purification Kit, K-3034–1, Bioneer Corporation, South Korea). MacrogenInc, (South Korea) sequenced the purified PCR products. All inter-transcribed spacer sequencing work was also performed by MacrogenInc, (South Korea) and was carried out on both strands of the submitted DNA fragments [121]. The purified PCR products were sequenced using an ABI 377 DNA. Auto-sequencer (PerkinElmer, Applied Biosystems Div., Waltham, USA) based on the same primers mentioned before.

#### In vitro evaluation of the antifungal activity of *Trichoderma sp.* against fungal pathogen

*Trichoderma* sp. was employed in an in vitro antagonistic assay to assess its biocontrol effects against the pathogenic fungus of barley by the dual culture technique [122]. Mycelial growth discs of 5 mm diam. removed under aseptic conditions from the growing edge of a 5 day-old pure culture of *Trichoderma* sp. and *Bipolaris* sp. were transferred and placed on the opposite of a Petri dish (9 cm) containing 20 mL of PDA and were kept 6 cm apart from each other. The plates were incubated for 7 days at 28°C, and the treatments were replicated in triplicate. Control dishes were cultures of *Bipolaris* sp. without the presence of *Trichoderma* sp. The growth of the pathogen and *Trichoderma* sp. was observed constantly, and radial growth was recorded by measuring the mean colony diameter on the 5th day of inoculation. The percent of inhibition (PI) of the test phytopathogenic fungus (*Bipolaris* sp.) was calculated using the following formula:

$$\mathbf{\%}\mathbf{P}\mathbf{I}=[({\mathbf{R}}_{1}-{\mathbf{R}}_{2})/{\mathbf{R}}_{1}]\times 100$$

R_1_ was the radial growth of the pathogen without *Trichoderma* sp., and R_2_ represents the radial growth of the pathogen inoculated with *Trichoderma* sp. [123].

#### Mycoparasitism

For microscopy examination, dual culture for both pathogenic and *Trichoderma* sp. was examined after 7 days of incubation, the regions where the hyphae of *Trichoderma* sp. and *Bipolaris* sp. interacted (the interaction zones) were observed. Characteristics used for differentiating between the hyphae of *Trichoderma* sp. and *Bipolaris* sp., were studied according to Rifai [117] and Wiese [118] using a light microscope (Leitz WETZLAR, Wetzlar, Germany).

#### Antagonistic effects of *Trichoderma sp.* culture filtrate (Antibiosis)

*Trichoderma* sp. was grown on PDA plates for 7 days at 28°C. A fungal disc of 5 mm in diam. of *Trichoderma* sp. mycelial growth was transferred from the periphery of mycelial growth and inoculated on Erlenmeyer flasks (250 mL) containing 100 mL of PDB. The flasks were then incubated in a shaking incubator at 150 rpm at 28°C. Mycelial growth was harvested on the 5th day of incubation. The culture was filtered through Whatman filter paper no. 1 and re-filtered through a Millipore syringe filter (0.22 μm).

A Petri dish (9 cm in diameter) containing 25 mL of PDA was supplied with 1 mL of *Bipolaris* sp. spore suspension and allowed to harden. After that, three wells with a diameter of 10 mm were made using a sterile cork-borer on each agar plate. The wells were subsequently filled with a cell-free culture of *Trichoderma* sp. (150 µL) using a well-diffusion technique described by Soliman et al. [124] with slight modifications. The plates were then placed in a refrigerator for 5h before being incubated at 28°C for 5 days. At the end of the incubation period, the inhibition zones were assessed.

#### *Biosynthesis and characterization of TiO*_*2*_* NP*

In our latest work, TiO_2_ NPs were prepared by an eco-friendly green synthesis method using titanium tetrachloride (TiCl_4_) solution and an aqueous extract of *Aloe vera* plant leaves, which were obtained from the Horticulture Department, Faculty of Agriculture, Zagazig University, Egypt after permission from Zagazig University. In brief, 100 mL of *A. vera* leaf extract was added dropwise to a 100 mL 1N TiCl_4_ solution in deionized water under continuous stirring. The pH of the mixture was adjusted to 9, and the stirring continued at room temperature for 4h. The formed NPs were filtered, washed with double-distilled water, and finally dried at 100°C overnight. The obtained dry powder was further calcined at 400°C for 4h. The prepared NPs were characterized by UV–Visible spectrophotometry, Fourier transform infrared (FTIR), X-ray diffraction (XRD), scanning electron microscopy (SEM), and transmission electron microscopy (TEM) as latest described [60].

#### TiO_2_ NPs suspension preparation and in vitro assessment of its antifungal activity and growth inhibition against *Bipolaris sp.*

Suspension of 50 mg/L of these NPs is prepared by dissolving NPs in distilled water and sonicating for 25–30 min using Elma (E15H Elmasonic) for equal distribution of NPs in water.

The antifungal activity of green synthesized TiO_2_ NPs was carried out using the well diffusion method [125]. One mL of *Bipolaris* sp. spore suspension prepared from 5-day-old culture was added to 25 mL of PDA and poured in Petri dish. After that, three wells with a 10 mm diam. were made. The wells were filled with 150 µL of two different concentrations of TiO_2_ NPs (25 and 50 mg/L) individually in triplicate. The culture plates were incubated at 28°C for 5 days, and the zones of inhibition were observed.

#### Inoculum preparation

PDA plates with 7-day-old pure cultures of *Trichoderma* sp. and *Bipolaris* sp. were used. After that, sterile water was added to the cultures, and the mycelium was scraped gently with a sterile glass rod. The spore suspension concentrations of *Trichoderma* sp. and *Bipolaris* sp. were adjusted to 2.5 × 10^7^ and 1.7 × 10^7^ conidia/ mL; respectively.

### In vivo experiments

#### Antagonistic activity of *Trichoderma sp. *on barley plants infected with *Bipolaris sp.* under filed conditions

##### Planting, growth conditions, and treatments

**Fig. 7:**
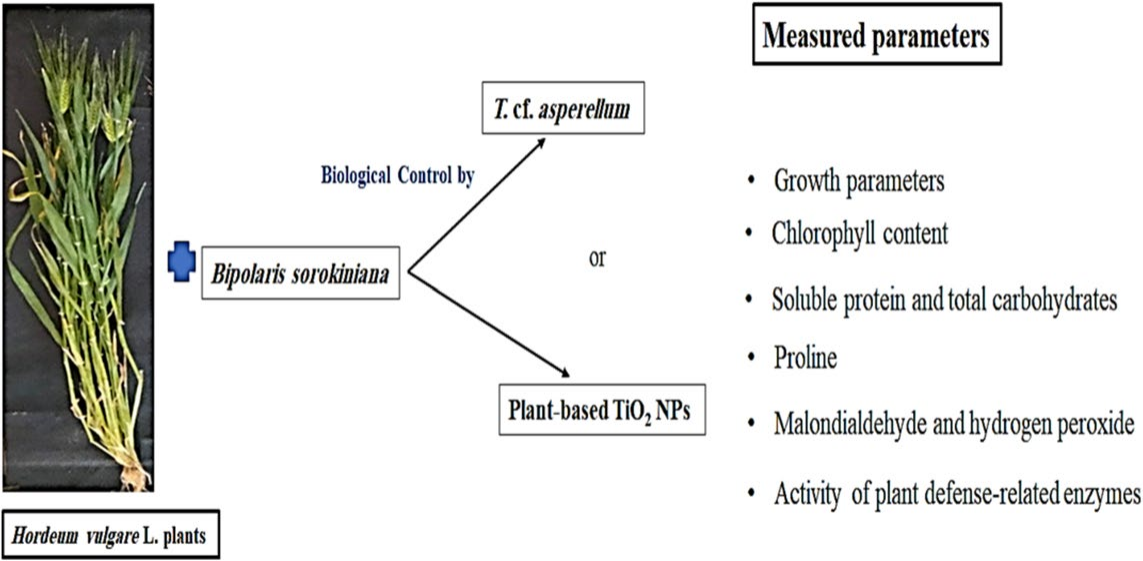
Schematic representation of the experimental set up

To assess *Trichoderma* sp.'s and the biosynthesized TiO_2_ NPs' capability for biocontrol against *Bipolaris* sp. on barley plants, a field experiment was carried out (Fig. 7). Barley (*Hordeum vulgare* L.) grains were acquired after permission from the Department of Plant Breeding and Genetics, Agricultural Research Center, Giza, Egypt, and were planted in the sandy clay soil of Zagazig, El-Sharkia Governorate. After one month of planting, a spore suspension of *Trichoderma* sp. was used as a foliar spray and drenched the soil near the stem region of *Trichoderma* sp.-treated plants. A concentration of 50 mg/L of TiO_2_ NPs was applied twice to the barley plant leaves, i.e. once before *Bipolaris* sp. application and then 7 days after inoculation. Ten days after *Trichoderma* sp. application, a spore suspension of *Bipolaris* sp. was used for infection by directly spraying the suspension using an atomizer on the leaves of the barley plants. Plants sprayed and irrigated with tap water only were used as a negative control. Six treatments were directed and arranged in a factorial design (2 × 3). Each treatment had 3 replicates. Four weeks after the *Bipolaris* sp. infection, disease symptoms appear. *Bipolaris* sp. was re-isolated from symptomatic tissues, and its identity was confirmed. The harvested samples were either immediately used or rapidly stored and frozen at − 20°C for further studies.

### Measurements

#### Growth traits

Morphological traits of barley plants were recorded 4 weeks after *Bipolaris* sp. infection. The following characteristics were measured for each treatment: shoot height (cm), fresh (fwt) and dry (dwt) weight of shoot (g), root, and spike. Three barley plants were collected for each treatment, and they were gently washed with flowing water to remove soil debris.

#### Measurement of physio-biochemical indexes

Fresh leaves of barley plants from each treatment were taken separately in order to measure the levels of enzymes linked to plant defense, proline, soluble protein, total carbohydrates, malondialdehyde (MDA), hydrogen peroxide (H_2_O_2_), and chlorophyll (Chl).

The quantitative analysis of Chl was done according to the method of Lichtenthaler and Wellburn [126] after the extraction of 100 mg of fresh leaves with acetone, and the absorbance was read at 644, 663, and 452.5 nm wavelengths, and its content was calculated by the following formula:

$$\mathrm{Chl\ }a (\mathrm{mg\ }{{\text{g}}}^{-1}\mathrm{ leaf\ fwt}) = [12.7\left({\text{OD}}663\right)- 2.69 ({\text{OD}}644)] \times \mathrm{ E}.{\text{V}}/1000 \times \mathrm{ fwt}$$

$$\mathrm{Chl\ }b (\mathrm{mg\ }{{\text{g}}}^{-1}\mathrm{ leaf\ fwt}) = [22.9({\text{OD}}644) - 4.68 ({\text{OD}}663)] \times \mathrm{ E}.{\text{V}}/1000 \times \mathrm{ fwt}$$

$$\mathrm{Carotenoids\ }(\mathrm{mg\ }{{\text{g}}}^{-1}\mathrm{ leaf\ fwt}) = (4.2\mathrm{ OD}452.5) - (0.0264\mathrm{ Chl}.\mathrm{\ a }+ 0.426\mathrm{ Chl}.\mathrm{\ b}) \times \mathrm{ E}.{\text{V}}/ (1000 \times {\text{fwt}})$$

*OD = Optical density, E.V = Extraction volume of sample, fwt = Fresh weight of sample.

The total soluble protein content of both infected and healthy barley leaves was measured [127] using a Folin–Ciocalteu reagent at 700 nm using bovine serum albumin as a standard. Fwt of barley leaves (250 mg) were ground in potassium phosphate buffer (50 mM pH 7.0) and centrifuged for 7 min at 6000 rpm (4°C) (MIKRO 200R Hettich Zentrifugen, Germany). Then 1 mL of the resultant supernatant was mixed with 5 mL of an alkaline copper solution. Total carbohydrate content was estimated by comparison with a glucose standard curve, as described by Dubois et al. [128]. Barley dried leaves (100 mg) were heated with 10 mL of 2.5N HCl in a boiling water bath for 3 h. The extract (0.1 mL) was then taken, and 1 mL of phenol was added. Sulfuric acid (2.5 mL) was added after 1 h of mixed properly, and the absorbance was measured at 490 nm.

The Bates et al. [129] method was used to estimate the proline content in barley leaves. In brief, 250 mg fwt of barley leaves were extracted and centrifuged in 3% sulphosalicylic acid. Then, 2 mL of filtrate was reacted with 2 mL of ninhydrin reagent and 2 mL of glacial acetic acid and then placed in a boiling water bath. Four mL of toluene were added, and the upper colored layer was separated, and its absorbance was read at 520 nm. Cell membrane decomposition of barley leaves was determined as the concentration of total 2-thiobarbituric acid (TBA) reactive ingredients [130]. To summarize, 250 mg of barley leaf tissues were extracted in 5 ml of 0.1% trichloroacetic acid (TCA). The supernatant (500 L), after being centrifuged for 10 min at 6000 rpm, was mixed with 2 mL of 20% TCA containing 0.5% TBA, incubated for 30 min at 95°C, and cooled on ice immediately. Absorbance at 532 and 600 nm was used for the calculation of the MDA equivalent. Based on the extinction coefficient of 155 mM^−1^ cm^−1^, the rates of lipid peroxidation were expressed as nmol g^−1^ fwt of the MDA-TBA complex formed. The MDA equivalent was calculated using the following equation:

$$\mathrm{MDA\ }({\text{nmol}}/\mathrm{ mL}) = [({\text{A}}532-{\text{A}}600)/155000]{10}^{6}$$

To quantify the H_2_O_2_ content in barley, leaf samples were collected from infected and treated plants. The reagent ferrous oxidation-xylenol orange was employed [131]. In 0.1% TCA, a known fwt of barley leaves (250 mg) was homogenized. Half mL of 100 mM potassium phosphate buffer (pH 6.8) and 2 mL reagent (1 M KI w/v) were added to 0.5 mL of the barley leaf extract. The reaction was left for 1 h in darkness, and the absorbance was measured at 390 nm.

### Plant defense-related enzyme determination

The concentrations of peroxidase (POX), catalase (CAT), phenylalanine ammonia-lyase (PAL), and lipoxygenase (LOX) were measured in order to confirm the effect of *T. cf. asperellum* and the biosynthesized TiO_2_ NPs against spot blotch disease in barley plants. To analyze the activities of POX and CAT, fwt of barley leaves (100 mg) were ground in 25 mL of potassium phosphate buffer (50 mM pH 7.0) and centrifuged at 6000 rpm (4°C) for 20 min. The supernatant was collected to measure the activities of POX and CAT, according to Bergmeyer [132] and Aebi [133].

The activity of PAL was determined in barley plant leaves [134] using phenylalanine as the substrate and the absorbance was recorded at 290 nm. According to Axelord et al. [135], the LOX activity was assessed using linoleic acid as a substrate. Linoleic acid (10 μL) and Tween 20 (50 μL) are both included in the test buffer, which is 10 mL of 0.1 M phosphate buffer with a pH of 9.0. By adding 0.1 mL of enzyme extract to 1 mL of freshly made assay buffer, the assay process was started. LOX activity was determined by observing the absorbance at 234 nm.

### Statistical analysis

The average and standard errors of 5 replicates (*n* = 5) are represented in the tables and graphed findings. The analysis of variance (ANOVA) was used to statistically confirm the results and by using Duncan's multiple range test (*p* < 0.05), it was concluded that there was a significant difference between the control and treatment groups. SPSS® 18.0 was used to perform the calculations. Using SPSS, Pearson's correlation coefficients (*r*) were carried out to understand the relationship between growth indices and different physio-biochemical parameters. Figures were assembled using OriginPro 8.5 for data analysis and graphing software.

## Conclusion

To achieve sustainable food production, agriculture must employ innovative strategies to decrease the use of agrochemicals. Consequently, NPs and fungal biological agents have been suggested as viable strategies with less environmental impact. Our research confirmed that *B. sorokiniana* causes spot blotch disease in barley plants, which results in morphological and physio-biochemical alterations. Nevertheless, on the host plant side, the application of *T.* cf*. asperellum* or green synthesized TiO_2_ NPs positively increased the host plant's tolerance against this pathogen and played an important role in the activation of a complex defense system that comprises: (1) induction of osmolytes such as proline, protein, and soluble sugars. (2) An increase in antioxidant defense-related enzyme production. All these defense systems neutralize the destructive effects of ROS, decreasing H_2_O_2_ and lipid peroxidation and maintaining homeostasis within *B. sorokiniana*-challenged barley plants. So, the application of *T.* cf. *asperellum* or green synthesized TiO_2_ NPs could be considered as an alternative or eco-friendly approach to protect barley plants from spot blotch disease caused by *B. sorokiniana*. Further studies with the *B. sorokiniana* pathogen must be carried out in the field to explore the potential of *T.* cf. *asperellum* and green synthesized TiO_2_ NPs combined applications as a viable strategy, which might be evaluated in the future to obtain better crop yields with less environmental impact, however further studies will be necessary to gain a comprehensive understanding of biological agent-NPs-plant interaction mechanisms.

## Data Availability

The relevant datasets supporting the results of this article are included within the article and the [GenBank NCBI] at: https://www.ncbi.nlm.nih.gov/nuccore/OP108262.1/*T.* cf.* asperellum.* https://www.ncbi.nlm.nih.gov/nuccore/OP714480.1/ *B. sorokiniana.*
